# On-chip integrated vertically aligned carbon nanotube based super- and pseudocapacitors

**DOI:** 10.1038/s41598-017-16604-x

**Published:** 2017-11-29

**Authors:** O. Pitkänen, T. Järvinen, H. Cheng, G. S. Lorite, A. Dombovari, L. Rieppo, S. Talapatra, H. M. Duong, G. Tóth, K. L. Juhász, Z. Kónya, A. Kukovecz, P. M. Ajayan, R. Vajtai, K. Kordás

**Affiliations:** 1 Microelectronics Research Unit, Faculty of Information Technology and Electrical Engineering, University of Oulu, P.O. Box 4500, FIN-90014 University of Oulu, Oulu, Finland; 2 Department of Mechanical Engineering, National University of Singapore, 9 Engineering Drive 1, EA-07-05, Singapore, 117575 Singapore; 3 Research Unit of Medical Imaging, Physics and Technology, Faculty of Medicine, University of Oulu, Oulu, Finland; 4 Department of Physics, Southern Illinois University, Carbondale, IL 62901 USA; 5 Department of Applied and Environmental Chemistry, and 5MTA-SZTE “Lendület” Porous Nanocomposites Research Group, University of Szeged, Rerrich B ter 1, H-6720 Szeged, Hungary; 6 Department of Applied and Environmental Chemistry, and 5MTA-SZTE Reaction Kinetics and Surface Chemistry Research Group, University of Szeged, Rerrich B ter 1, H-6720 Szeged, Hungary; 7 Department of Material Science and NanoEngineering, Rice University, Houston, Texas 77005 United States

## Abstract

On-chip energy storage and management will have transformative impacts in developing advanced electronic platforms with built-in energy needs for operation of integrated circuits driving a microprocessor. Though success in growing stand-alone energy storage elements such as electrochemical capacitors (super and pseusocapacitors) on a variety of substrates is a promising step towards this direction. In this work, on-chip energy storage is demonstrated using architectures of highly aligned vertical carbon nanotubes (CNTs) acting as supercapacitors, capable of providing large device capacitances. The efficiency of these structures is further increased by incorporating electrochemically active nanoparticles such as MnO_x_ to form pseudocapacitive architectures thus enhancing device capacitance areal specific capacitance of 37 mF/cm^2^. The demonstrated on-chip integration is up and down-scalable, compatible with standard CMOS processes, and offers lightweight energy storage what is vital for portable and autonomous device operation with numerous advantages as compared to electronics built from discrete components.

## Introduction

In order to keep pace with Moore’s law and drive advantages of miniaturization to the next stage of evolution, it is becoming clear that techniques for heterogeneous integration of a multitude of devices on a single chip need to be developed^1,2^. Along with the need for heterogeneous integration rapid, reliable and resilient energy sources needed to operate these with low power consumption takes the forefront of research and development. Among several available energy sources, the fast charge and discharge capabilities of supercapacitors and pseudocapacitors make them ideal for powering such devices. Supercapacitors allow for rapid energy storage and deliveries compared to batteries and are more cost effective. Today’s largest commercial supercapacitors have capacities up to several thousands of Farads that are applied in hybrid cars as energy storage units in various kinetic energy recovery and start-stop systems^3,4^. Smaller storage devices with a typical capacity of ~1 F are ubiquitous in portable and other autonomous electrical devices that serve as back-up or uninterruptable power systems, small battery replacements etc.

From past investigations, it has been well established that the low density, porous microstructure, excellent intrinsic and interfacial electrical transport properties of carbon-based nanostructures renders them to be the ultimate choice for electrode materials in supercapacitors^5–8^. Further, simple modifications of the carbon surfaces, for example, attaching electrochemically active moieties, such as various phases of spinels (M^2+^M^3+^_2_O_4_)^9–11^, ZnO^12^, RuO_2_·*n*H_2_O^13^, Ni(OH)_2_^14^ and MnO_2_^15^ on the surface of carbonaceous materials hybrid structures utilizing pseudocapacitive behavior or faradaic redox-peaks and having approximately an order of magnitude increase in capacitance compared to the corresponding supercapacitors can be achieved^16–18^. Depending on the electrolyte used pseudocapacitors are capable of storing very high energy densities, ~5000 J·g^−1^ or ~1.5 W·h·g^−1^ ^16–18^, which in some cases even competes with that of Li-ion batteries. Even though successful fabrication of high energy and power density supercapacitor is achieved in the past several decades, their reliable integration into solid and flexible chips and/or packages, remain as one of the major challenges. Overcoming this challenge is critical in order to remove the technology bottleneck this might pose for heterogeneous integration of on-chip power sources for a variety of applications. The seamless direct growth of the electrode materials on conductive substrates would offer a number of practical advantages that point beyond today’s individual capacitor components used especially in portable electrical devices. On the one hand, on-chip and in-package integrated capacitors can lead to smaller and more compact electronics than that built from discrete components, which inevitably results in a progress of increased device functionality in the future in accordance with the growing demands of semiconductor industry projected by the 2015 International Technology Roadmap for Semiconductors^19^. On the other hand, integrated electrode structures will also contribute to better functional reliability and device safety^20–22^, both of which are extremely important from practical point of view.

Recent advances on super and pseudocapacitors having nanostructured carbon based electrodes integrated on flexible substrates^23–32^ and rigid Si^23,25,33–37^ chips have proven the viability of the on-chip energy storage concept while other support materials for pseudocapacitive materials such as conductive polymers^38–41^ have also gathered much interest. Though some of the latest pseudocapacitor electrode materials have reported to have areal capacitances over 1 F/cm^2^ the integration of these materials still have challenges for on-chip integration^42,43^.

In electrical applications of on-device CNT forests, it is essential to ensure a seamless interface between the nanotubes and the metallic current collector layer of the device. In our previous study, we confirmed that carbon nanotubes can be grown on a number of different metal foils and slabs when using aluminum buffer layer without significantly increase in the series resistance of the metal-CNT interface in supercapacitor applications^44^. In our current effort, we implement our well-tested growth technology to synthesize interdigitated micro patterns of aligned CNT forests directly on a silicon chip. We show well-aligned carbon nanotubes grown directly on Pt current collector on Si template functionalized with electrochemically active MnO_x_ nanoparticles allowing for on-chip pseudocapacitor devices with a large storage capacity of 8.8 mF with a specific areal capacitance of 37 mF/cm^2^. The process, which utilizes simple CVD technique for CNT growth on electrically conductive micropatterned diffusion barrier and catalyst layers, is scalable, robust and CMOS compatible. The proposed technology paves the road towards devices of ultra-large storage capacity with a great potential to be used in e.g. vehicles, grids, and electronics^1,45^.

**Figure 1:**
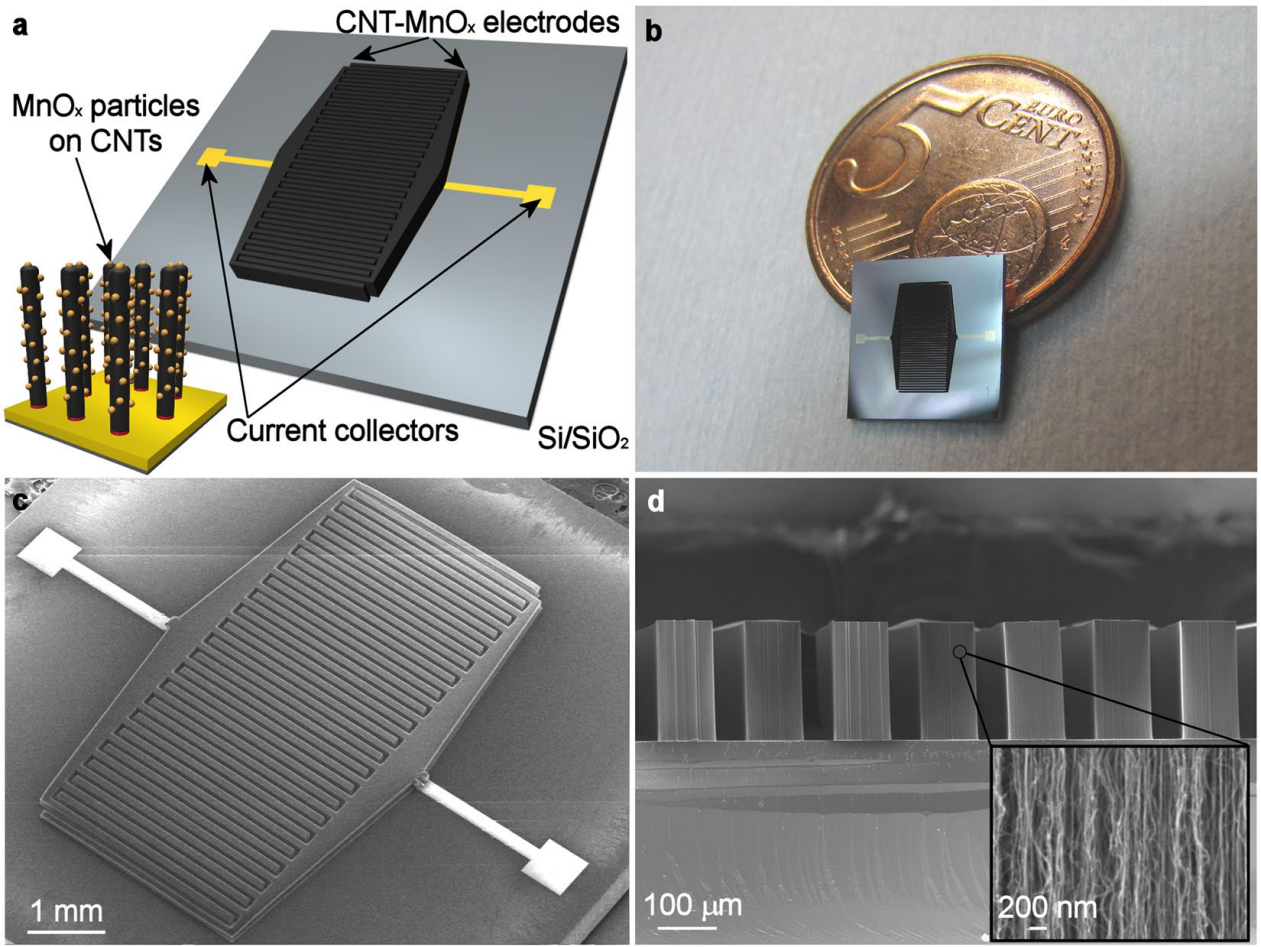
Structure of the planar interdigitated structure used as on-chip super and pseudocapacitor. (**a**) Schematic drawing of the planar on-chip interdigitated electrode structures with inset describing the structure of the current collector-CNT-MnO_x_ nanoparticle interface. (**b**) Optical camera image illustrating the size of the synthesized CNT structure on Si/SiO_2_ chip. (**c**) FESEM image of the capacitor structure showing interdigital structure of the CNT forest after synthesis. (**d**) Cross section FESEM image of the aligned CNT forest digits. Inset is a close up image of the aligned parallel nanotubes in the film.

Pt and Mo thin film metallization layers were studied to reveal their feasibility as conductive current collector layer under the diffusion barrier Al and catalyst Fe layers. While Mo exhibited slightly better CNT forest growth and better adhesion onto the substrate, it was found to be oxidized by the permanganate during the deposition of manganese oxides. This rendered Mo to be less appropriate for robust growth and stability of the CNTs that is needed for this application. However, Pt was found to be chemically and structurally stable. Furthermore, Pt metallization is also preferred since it allows proper soldering/bonding and integration with other electrical circuits and packages. This makes Pt a reasonable material of choice for current collector. The schematic structure (Fig. 1a) of the planar CNT-MnO_x_ capacitor illustrates interdigits of vertically aligned CNT electrodes and the carbon nanotubes with MnO_x_ nanoparticles anchored on them. The CNT forests are directly grown onto conductive metal layer. The part of the metal layer also acts as connector pads. During the growth it was found that though it was possible to synthesize the CNT forests with lengths of the CNTs ~500 µm long, the outermost digits of the interdigitated capacitor structure had an increasing tendency to bend thus shorting the capacitor once the length of the CNT forests started to go over 300 µm. Because of this, the CNT forest length investigated in this study was fixed to 200 µm. Optical (Fig. 1b) and FESEM (Fig. 1c) images show the interdigitated structure with a digit width and spacing of 100 µm and 50 µm, respectively. It can be seen from the cross section image (Fig. 1d) that the CNTs are also well aligned within the structure. One major limiting factor of super and pseudocapacitors performance is the diffusion/drift distance of ions in present in the electrolyte, which is quite optimal with interdigitated structures because of the proximity of opposite electrodes. The well-defined spatial separation of the electrodes on the same chip without needing a separator layer inherently simplifies the typical sandwich type structures in conventional devices and improves ion transport since this structure of devoid of any porous separator. Aqueous based Na_2_SO_4_ was chosen as an electrolyte, as it has been widely used in super- and pseudocapacitor research and is non-corrosive and non-toxic and also provides good electrical conductivity and 1 V operational window.

**Figure 2:**
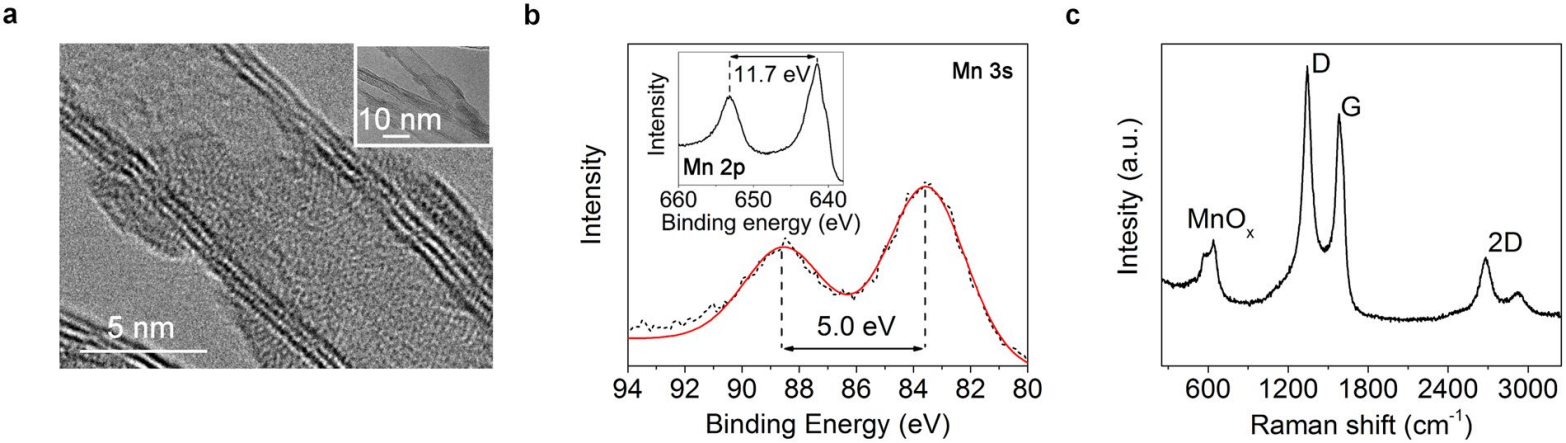
Structure and chemical composition of the nanocomposite. (**a**) A high resolution TEM image of MnO_x_ on the surface of a CNT. (**b**) X-ray photoelectron spectra of the Mn 3 s and Mn 2p regions (inset). (**c**) Raman spectrum of the CNT-MnO_x_ composite.

The size of the MnO_x_ particles on the surface of the CNTs varies (Supplementary Fig. S1). TEM image in Fig. 2a shows that the MnO_x_ attached to the CNTs has really low crystallinity and the particles are of size  between 2 and 8 nm. The carbon nanotube diameter varies from 7 to 10 nm (3 to 5 walls) (Supplementary Fig S2a). The low crystallinity is also supported by the electron diffraction pattern (Supplementary Fig S2b). The Mn/C atomic ratio determined by EDX is ~0.12 and comparison of TEM light field and dark field images indicates the MnO_x_ covering CNTs (Supplementary Fig S3). The 5.0 eV splitting of Mn 3 s XPS peak displayed in Fig. 2b indicates an average oxidation number of 3.4 for the manganese ion suggesting a mixture of manganese oxides with oxidation states 4 and 3 such as MnO_2_ and Mn_2_O_3_, respectively^46^. On the other hand, the spin energy separation of 11.7 eV between Mn 2p_3/2_ and Mn 2p_1/2_ peaks is in agreement with previously reported values for MnO_2_^47,48^. Also the Mn 2p_3/2_ peak has a shoulder which is characteristic for MnO_2_^46^.

Raman spectrum (Fig. 2c) measured from the top of MnO_x_ decorated CNT forests shows a high I_d_/I_g_ peak ratio of 1.20 which indicates large amount of defects in the graphitic lattice of the carbon nanotubes^49^, likely caused be the high growth rate of the nanotubes (~200 µm/min). The 2D peak at ~2700 cm^−1^ which is common for graphene and single to few walled nanotubes is also clearly visible. The wide peak area between wavenumbers 570 and 650 cm^−1^ supports the low crystallinity and also suggests the presence of several manganese oxide phases^41^ with α-MnO_2_ being the most dominant^50,51^.

**Figure 3:**
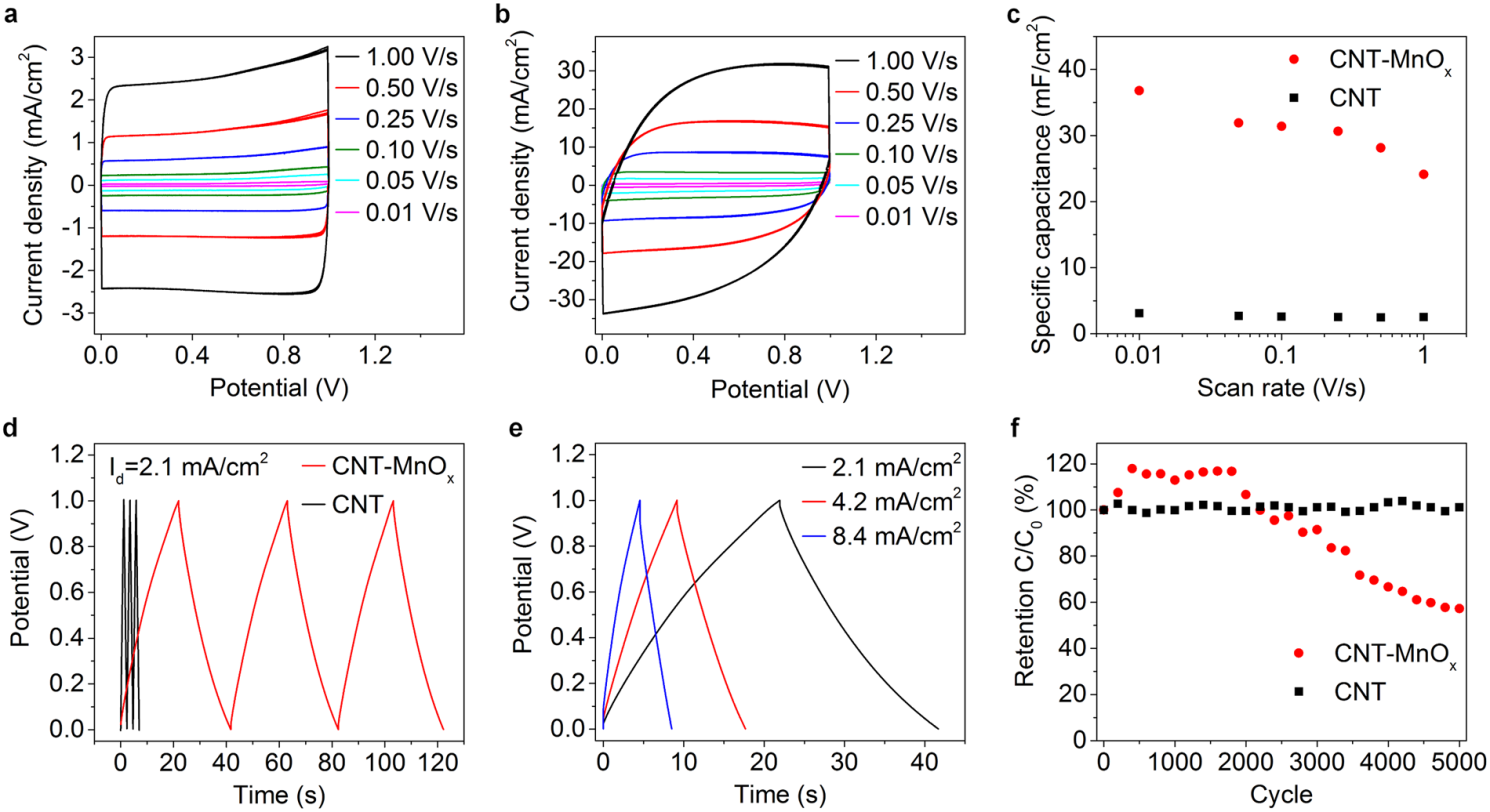
Electrochemical behavior of the on-chip capacitor devices. (**a**) Cyclic voltammetry curves of CNT supercapacitor. (**b**) Cyclic voltammetry curves of CNT-MnO_x_ pseudocapacitor. (**c**) Corresponding calculated specific capacitances at different scanning rates. (**d**) Charge-discharge curves of CNT and CNT-MnO_x_ measured at 2.1 mA/cm^2^ current density. (**e**) Charge-discharge curves of a CNT-MnO_x_ pseudocapacitor at different current densities. (**f**) Retention of both types of capacitors over 5000 cycles.

For electrochemical characterization of the devices cyclic voltammetry curves were measured using scanning rates between 10 mV/s to 1 V/s. (see Supplementary information for the calculations). The CV curves of CNT capacitor (Fig. 3a) have near ideal rectangular shapes and the measured capacitance varies a little with different scanning rates having a specific capacitance of 2.6 mF/cm^2^ for all scanning rates except for the slowest scan at 10 mV/s having a value of 3.1 mF/cm^2^. The values are ~6 times higher than reported by Jiang *et al*.^35^. This is likely due to the longer length of the CNT forest and smaller CNT diameter compared to the previous study which would lead to larger number of CNTs and larger active surface area. The capacitance of the CNT-MnO_x_ capacitors is more limited by slower redox reactions of MnO_x_ compared to the double layer charge formation on the CNTs, which can be seen as rounded edges in the CV-curves (Fig. 3b). The capacitance using 10 mV/s was measured to be 8.8 mF which results in a specific capacitance of 37 mF/cm^2^ that drops at higher scan rates (Fig. 3c) which is a typical signature for pseudocapacitive redox reactions of MnO_x_. Still, even measured at 1 V/s rate, the capacitance is ~10 times higher than that of the CNT capacitor (24 mF/cm^2^ vs 2.5 mF/cm^2^). It is worth noting here that an increased MnO_x_ loading, in principle, could increase further the absolute and hence the specific capacitance of the devices, however with a price of additional internal series resistance and less ideal device operation reducing the power performance of the capacitor^27,52,53^. This could be seen with samples that had less MnO_x_. In these samples the capacitance is less influenced by the slow redox reaction of the MnO_x_, but the overall capacitance is lower (Supplementary information Fig. S4).

The capacitance was also calculated from charge-discharge measurements (see Supplementary information for the calculations). The measurements give similar results to CV measurements except for the lowest current density applied resulting in even higher capacitance (48 mF/cm^2^ at 0.2 mA/cm^2^) than that calculated from the corresponding CV curves (Fig. 3d and e). The retention of the capacitors was also tested. (Fig. 3f). The CNT capacitor had steadily 100% of its original capacitance, whereas the capacitance for CNT-MnO_x_ first increased to ~120% during the first 400 cycles but then starts decaying after ~2000 cycles to ~60% of the original value at 5000 cycles probably due to microstructural defects in the finger structure or due to poor adhesion of MnO_x_ particles on the CNT surface. Thus the retention performance of the CNT-MnOx device could be improved by depositing the MnO_x_ for example electrochemically^37^ which would in principle provide better adhesion of the pseudocapacitive material.

**Figure 4:**
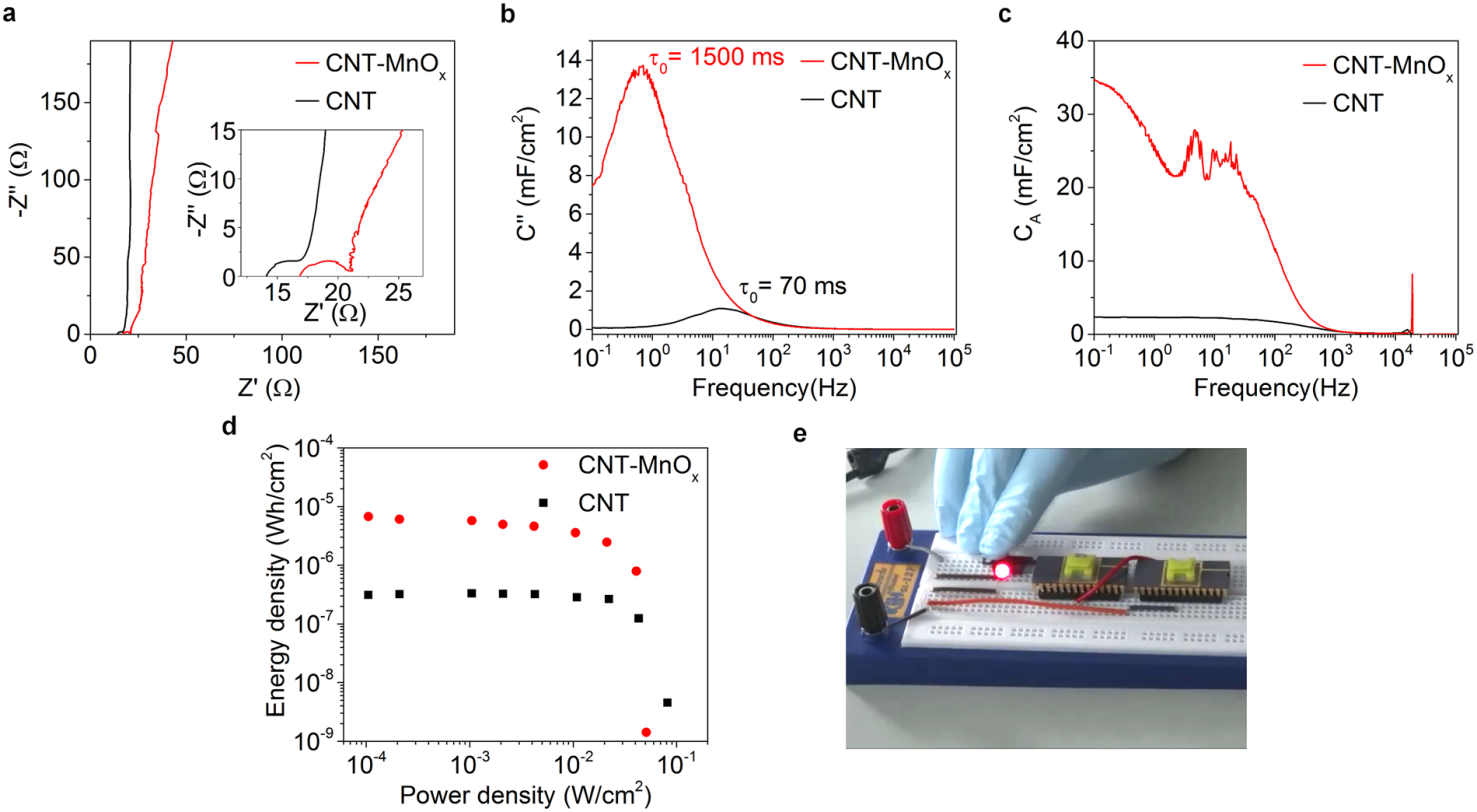
Electrochemical impedance spectrometry measurements, Ragone plots and practical testing. (**a**) Nyquist plots of impedance. The inset shows the high frequency region in more detail. (**b**) Imaginary part of the capacitance and the time constants. (**c**) Frequency response for capacitance for both devices. (**d**) Ragone plots of specific power density and energy density. (**e**) Lighting of an LED with two on-chip capacitor devices mounted in hybrid ceramic-plastic packages.

In order to get further insight of the devices formed, Electrochemical Impedance Spectroscopy (EIS) measurements were carried out in a frequency range from 100 mHz to 100 kHz. From the Nyquist diagrams (Fig. 4a) it can be seen that the impedance of the CNT-MnO_x_ capacitor is more dependent on the frequency compared to the CNT capacitor. The equivalent series resistance (ESR) is found to be 14 Ω for CNT capacitor and 17 Ω for CNT-MnO_x_ capacitor. ESR resistance is the sum of contact resistance between the current collector and the electrode material, electrolytic resistance in the porous electrode structure and resistance of the metal oxide^27,33^. The semicircle is interpreted as parallel resistance within electrode-electrolyte interface caused by the charge transfer resistance (3 Ω for CNT and 4 Ω for the CNT-MnO_x_ capacitor). The imaginary part of the capacitance versus frequency *C*_*A*_ *”*(*f*) in Fig. 4b, corresponds to the energy losses in the capacitor (see Supplementary information for calculation). The relaxation time constants *τ*_0_ (i.e. the minimum time to discharge the stored energy in the capacitor) are 70 ms for CNT and 1500 ms for CNT-MnO_x_ capacitor (reciprocal of the response frequency *f*_0_ at the maximum imaginary capacitance *C*_*A*_”). These values are rather short despite the quite high capacitances of our devices because of the overall small internal resistances of the devices thus the capacitors have the ability for fast discharge thus having a high power capability. The frequency response of capacitance shown in Fig. 4c (see Supplementary information for calculation) indicates our on-chip supercapacitor may be operated up to 120 Hz (at −3dB point), whereas the pseudocapacitor functions ideally in DC operation. (It is worth noting that both types of electrode materials have capacitances close to the corresponding values measured by using cyclic voltammetry).

The energy and power densities obtained from the charge-discharge measurements indicate the operational range for both devices as displayed in the Ragone plot in Fig. 4d (see Supplementary information for calculation). The calculated maximum energy densities are 0.3 µWh/cm^2^ and 6.7 µWh/cm^2^, whereas the maximum measured power densities are 81.8 mW/cm^2^ and 51.0 mW/cm^2^ for the CNT and CNT-MnO_x_ capacitors, respectively. The theoretical power density *P*_*d*_ for the CNT and CNT-MnO_x_ capacitors (74.2 mW/cm^2^ and 62.2 mW/cm^2^, respectively) are close to the measured values (see Supplementary information for calculation). The energy density drops faster with higher power in CNT-MnO_x_ capacitors due to the relatively slow redox reactions on the MnO_x_ surface in contrast with the formation of the double layer charge at the interface of CNTs and electrolyte in supercapacitors. The areal, volumetric and gravimetric performance values of the devices are listed in Supplementary Table S1 and are in the same scale as most of the previously reported research^24–28,31–35,37–39^. It should be noted that using ionic liquids as electrolyte would allow for ~3 V operation range which would in principle increase both energy and power densities by an order of magnitude^18,33^.

In order to demonstrate the immediate applicability of the on-chip energy storage system, they were utilized as sources to power a regular light emitting diode. For this purpose, supercapacitor chips were cased with 3D-printed plastic compartments to accommodate the electrolyte in a reliable manner. The chips were wire bonded to dual-in-line packages to produce easy-to-mount devices and then the plastic cases were filled with the electrolyte. Two of the devices were then mounted on a test board and connected in series to increase the operational voltage range to 2 V suitable for flashing an ordinary light-emitting diode (Fig. 4e).

In conclusion, we successfully demonstrated on-chip super and pseudocapacitor devices with vertically aligned carbon nanotubes and CNT-MnO_x_ nanocomposites having low crystalline MnO_2_ and Mn_2_O_3_ as dominant oxide phases. Our approach to grow lithographically defined micropatterns of vertically aligned carbon nanotubes on conductive metals using Al buffer layer proved to produce good electrical contact with the on-chip collector. The obtained on-chip electrode structures were suitable for subsequent chemical deposition of MnO_x_ to significantly improve the electrochemical storage capacity of the devices. The specific capacitance 37 mF/cm^2^ and energy density 6.7 µWh/cm^2^ measured for the CNT-MnO_x_ devices and the scalable CMOS compatible technology suggest the chips to be implemented in various energy storage systems. The demonstrated small planar device with high capacitance (8.8 mF), rapid and efficient charging/discharging capabilities as well as the easy to integrate platform offers an attractive energy management solution to e.g. IoT and mobile electronics and to complement energy scavenging units where the capacitors can be implemented to replace or combine with batteries. Our work proves practical viability of the on-chip integrated storage devices whose operation voltage window and thus energy storage capacity may be increased even further for instance by implementing ionic liquids as electrolytes, optimizing MnO_x_ load and the microstructure of the electrodes as well as by using other promising electrochemically active materials in conjunction with the on-chip CNT films.

## Materials and Methods

The planar capacitor patterns of 100 µm linewidth and 50 µm spacing were done using a standard photolithography process and a lift-off method on 4″ Si wafer with 1.5 µm thermal oxide. Two types of electrode materials platinum and molybdenum were used. A 10 nm Ti adhesion layer was sputtered on Si/SiO_2_ substrate followed by 200 nm of Pt or Mo conductive layer as a current collector, a 10 nm of Al diffusion barrier layer and ~1.1 nm Fe catalyst layer for CNT synthesis. After the deposition and lift-off, the wafer was cut into pieces of 10 × 10 mm.

Carbon nanotubes were synthesized in a cold wall low pressure chemical vapor deposition reactor equipped with 2″ graphite heater (Aixtron, Black Magic, UK). The chamber was first evacuated to 0.2 mbar base pressure and then filled with process gases of H_2_, N_2_ and C_2_H_2_ to 500 mbar chamber pressure, having gas flows rates of 700 sccm, 500 sccm, and 10 sccm respectively. The substrates were then rapidly heated to 670 °C keeping the gas flows and chamber pressure constant for 1 minute resulting in ~200 µm long CNT forests. After the CNT synthesis the nanotubes were carefully wiped off from the contact pads and conductor wires thus leaving one CNT covered electrode having an area of 8.56 mm^2^ and the total device having an area of 23.9 mm^2^ including the gaps between the electrodes.

For MnO_x_ deposition the Si chips with aligned CNTs were first soaked in acetone to properly wet the entire surface and to allow proper permeation of the liquid into the intertubular pores of the nanotube forests. After 30 minutes, the chips were flushed with de-ionized (DI) water, and placed in 10 mL solution of 0.1 M KMnO_4_ (Sigma Aldrich) for 30 min at 70 °C to speed up the natural decomposition of the peroxide into various forms of manganese oxides. At last, the samples were rinsed with DI water again.

Electrochemical measurements on the pristine and manganese oxide decorated electrodes were carried out in 1 M Na_2_SO_4_. To reduce surface tension and improve surface wetting with the aqueous electrolyte, 5 mM of Triton X-100 surfactant was added to the electrolyte. The capacitor devices were contacted under a probe station and connected to a potentiostat-galvanostat. (Princeton Applied Research VersaSTAT 3). The electrochemical performance of the planar capacitors was assessed by cyclic voltammetry (CV), charge-discharge and electrochemical impedance spectroscopy (EIS) measurements. The structure and chemical composition of the grown materials were assessed by the means of field emission scanning electron microscopy (FESEM, Zeiss Ultra Plus and Oxford Instruments EDX detector installed on the device) transmission electron microscopy (TEM, Tecnai G2 20 X-Twin microscope with a tungsten thermionic cathode operated at an accelerating voltage of 200 kV and JEOL JEM-2200FS EFTEM/STEM 200 kV), X-ray photoelectron spectroscopy (XPS, Thermo Fisher Scientific Escalab 250 XI system with Al Kα X-ray source, 1486.6 eV, data evaluation using Avantage software) and Raman spectroscopy (Thermo Scientific DXR2xi, λ = 532 nm).

## Electronic supplementary material

Supplementary information
