# Supplementary material for: On-chip integrated vertically aligned carbon nanotube based super- and pseudocapacitors
Source: Sci Rep. 2017 Nov 29;7:16594. doi: 10.1038/s41598-017-16604-x (PMC5707404; doi:10.1038/s41598-017-16604-x)
Supplement: Supplementary file 1 — Supplementary information [file 41598_2017_16604_MOESM1_ESM.pdf]

Supplementary Information

On-chip integrated vertically aligned carbon nanotube based super- and pseudocapacitors

O. Pitkänen, T. Järvinen, H. Cheng, G. S. Lorite, A. Dombovari, L. Rieppo, S. Talapatra, H.M. Duong, G. Tóth, K.L. Juhász, Z. Kónya, A. Kukovecz, P.M. Ajayan, R. Vajtai and K. Kordás

Materials characterization

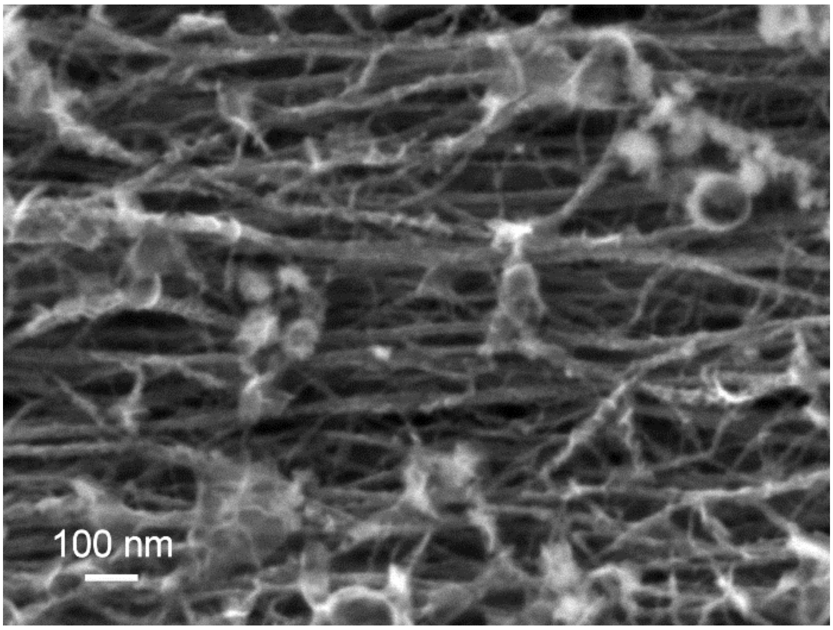

Figure S1 High resolution SEM image of CNT-MnO_x.

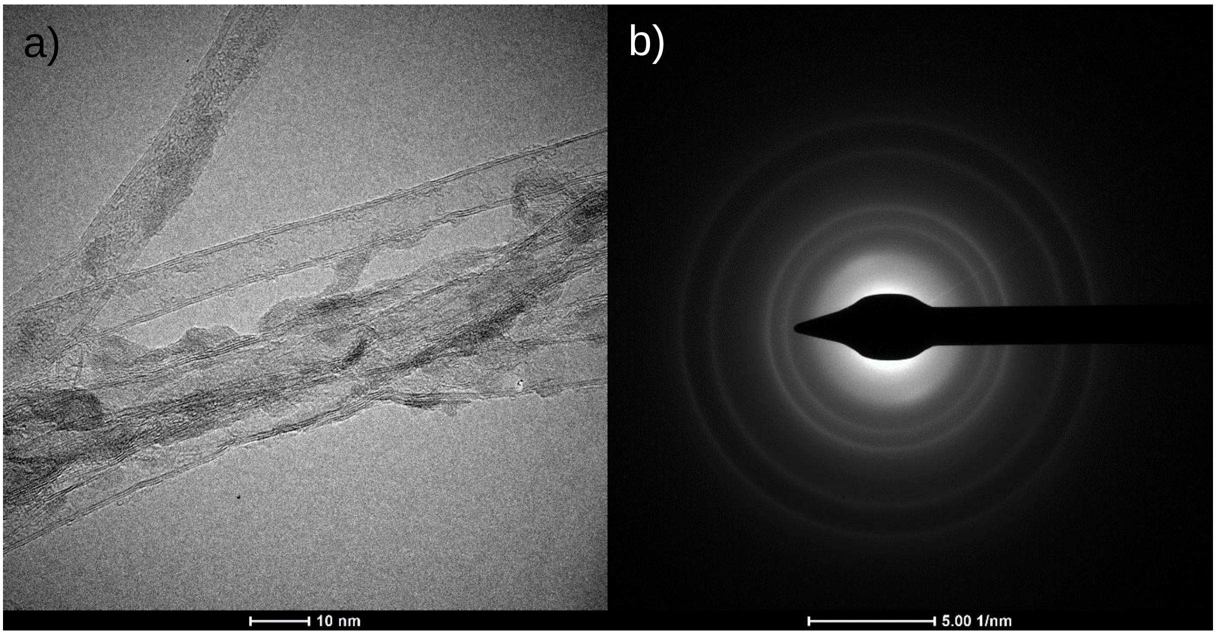

Figure S2 a) TEM image of the images of the MnO_x particles on CNTs and **b)** Electron diffraction pattern of the CNT-MnO_x

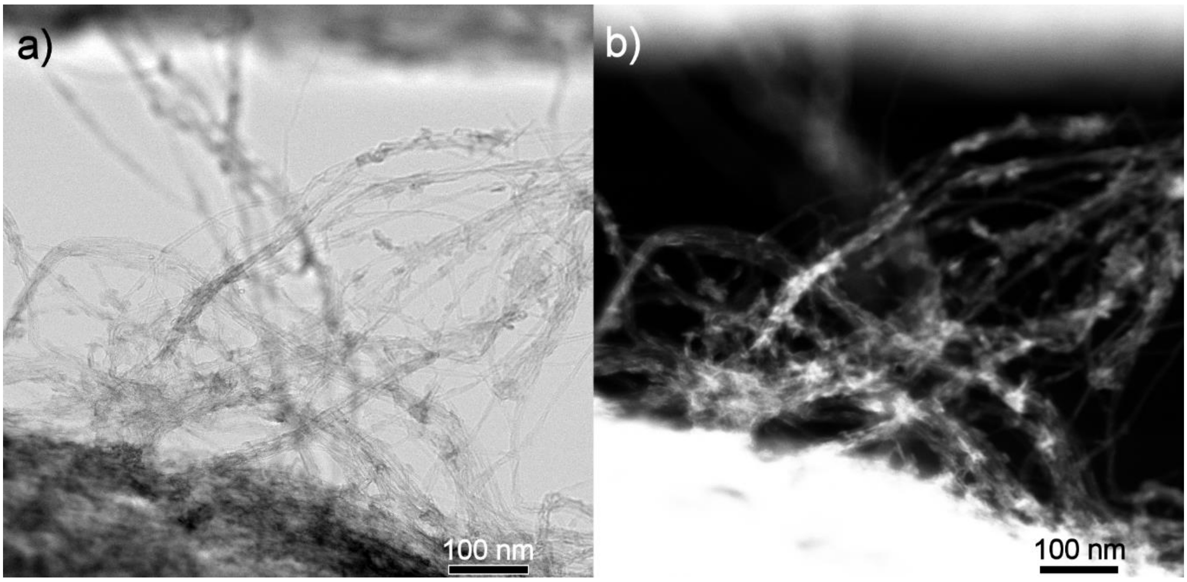

Figure S3 a) Bright and **b)** dark field TEM images of the CNT-MnO_x

Electrochemical measurements

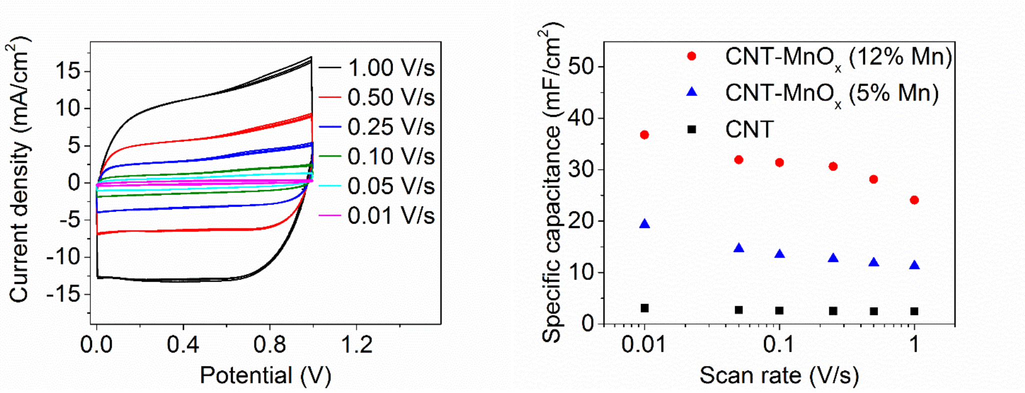

Figure S4 Cyclic voltammetry curves of CNT-MnO_x pseudocapacitor with 5% Mn/C ratio and specific capacitance comparison with two different Mn loadings and without MnO_x.

Calculation of the capacitance

The specific capacitances C_A were calculated from the averages of the integrated current-time hysteresis curves normalized by the electrode area $C_A = \frac{\int_{V_1}^{V_2} I(V)dV}{dV/dt \cdot \Delta U \cdot A}$, where $I(V)$ is the charging current, dV/dt is the scan rate, ΔU is the used voltage range between V_1 and V_2 and A is the area of the device. The capacitance was calculated from charge-discharge measurements with $C_A = \frac{I}{\Delta V/\Delta t \cdot A}$, where I is the used current, ΔV the voltage window, Δt is the discharge time and A is the area of the capacitor.

Calculation of the relaxation time constant and frequency response

The imaginary part of the capacitance versus frequency $C_A''(f)$ (Fig. 4b) corresponds to the energy losses in the capacitor and calculated as $C_A''(f) = \frac{Z'(f)}{2\pi f |Z(f)|^2 \cdot A}$, where f is the frequency, Z' the real part of the impedance, $|Z(f)|$ is the absolute value of the impedance and A is the area of the capacitor. The relaxation time constant τ_0 (i.e. the minimum time to discharge the stored energy in the capacitor) can be derived from the response frequency f_0 of the maximum imaginary capacitance C_A'' using $\tau_0 = \frac{1}{f_0}$. The frequency response of capacitance (Fig. 4c) can be estimated as $C_A = -\frac{1}{2\pi f Z''(f) \cdot A}$, where Z'' is the imaginary part of the impedance.[1]

Calculation of the power and energy densities

Energy density values of the on-chip devices are obtained from $E_d = \frac{CV^2}{2A}$, where C is the calculated capacitance from charge-discharge measurements, V is the used voltage range and A is the area of the device. The power density is calculated using the equation $P_d = \frac{E_d}{\Delta t}$, where E_d is the calculated energy density from charge-discharge measurements and Δt is the discharge time. The theoretical power density P_d is calculated as $P_d = \frac{V^2}{4\, R_{ESR} \cdot A}$, where V is the voltage range, R_{ESR} the equivalent series resistance and A is the area of the device.[1-3]

Table S1. Performance of the devices normalized by area, volume and mass

	**CNT**	**CNT-MnO$_x$**
Device capacitance [mF]	0.7	8.8
Areal capacitance [mF/cm^2]	3.1	37
Volumetric capacitance [F/cm^3]	0.2	1.8
Gravimetric capacitance [F/g]	12	64
Device energy [uWh]	0.1	1.6
Areal energy density [uWh/cm^2]	0.3	6.7
Volumetric energy density [uWh/cm^3]	16	335
Gravimetric power density [mWh/g]	1.2	12
Device power [mW]	20	12
Areal power density [mW/cm^2]	82	51
Volumetric power density [W/cm^3]	4.1	2.6
Gravimetric power density [W/g]	319	89

References

1. Yoo J. J. *et al.* Ultrathin Planar Graphene Supercapacitors. *Nano Lett.* 2011, **11,** 1423-1427.
2. Pech D. *et al.* Ultrahigh-power micrometre-sized supercapacitors based on onion-like carbon. *Nat. Nanotechnol.* 2010, **5,** 651-654.
3. El-Kady M. F., Kaner R. B. Scalable fabrication of high-power graphene micro-supercapacitors for flexible and on-chip energy storage. *Nat. Commun.* 2013, **4,** 1475.
